# Individuals’ Acceptance to Free-Floating Electric Carsharing Mode: A Web-Based Survey in China

**DOI:** 10.3390/ijerph14050476

**Published:** 2017-05-02

**Authors:** Yun Wang, Xuedong Yan, Yu Zhou, Qingwan Xue, Li Sun

**Affiliations:** 1 MOE Key Laboratory for Urban Transportation, Complex System Theory and Technology, School of Traffic and Transportation, Beijing Jiaotong University, Beijing 100044, China; 13114248@bjtu.edu.cn (Y.W.); 14114231@bjtu.edu.cn (Y.Z.); 14114258@bjtu.edu.cn (Q.X.); 2 School of Architecture and Urban Planning, Beijing University of Civil Engineering and Architecture, No.1 Zhanlanguan Road, Xicheng District, Beijing 100044, China; sunli@bucea.edu.cn

**Keywords:** carsharing, battery electric vehicles, private car ownership, hierarchical tree-based regression, policy implications

## Abstract

Carsharing is growing rapidly in popularity worldwide. When the vehicles involved are Battery Electric Vehicles (BEV), carsharing has been proven to remarkably contribute to easing energy and environment crises. In this study, individuals’ acceptance to carsharing in China was measured from three aspects: carsharing mode choice behavior, highest acceptable price to use carsharing, and willingness to forgo car purchases. The data were collected by a web-based survey. The hierarchical tree-based regression (HTBR) method was applied to explore the effects of potential influencing factors on individuals’ acceptance, and some interesting findings were obtained: participants who know about carsharing were more likely to use carsharing, pay higher prices and forgo car purchases; the most competitive trip purpose and trip distance for choosing carsharing were, respectively, business activities and 11–20 km; most participants (47.1%) were willing to pay 1–2 Yuan per minute to use carsharing, and males or participants with higher income-level could accept higher price; and when car purchase restrain policy (CPRP) was carried out in a city or the urban public transport service level (UPTSL) was high, participants were more willing to forgo car purchases. Based on the above findings, corresponding policies were proposed to provide guidance for successful establishment of carsharing in China.

## 1. Introduction

The exorbitance of automobile usage and its negative impacts on the energy and environment have been a major global issue and attract growing concerns. Ahmad and Oliveira [1] pointed that about 23% of energy-related CO_2_ emissions were emitted by the transportation sector. Moreover, the emissions from private cars accounted for nearly half of the whole transportation sector. Therefore, numerous countries, such as USA, Australia, United Kingdom, Switzerland, Germany, Japan, China, Korea, etc., are gradually involved in the efforts to relieve CO_2_ emissions. It has been proven that emissions from the transportation sector can be reduced by establishing reasonable policies for a more sustainable transportation system [2].

Promoting alternative fuel vehicles, such as hybrid and electric vehicles, offers an efficient pathway to reduce CO_2_ emissions from private cars. As an innovative and environmentally-friendly transportation mode, carsharing, i.e., a mode where individuals can rent cars for a short time period, has been proven to have an obvious effect on the private car reduction [3]. Carsharing has prominent advantages over the private car in the following four aspects: Firstly, the emergence of carsharing makes remarkable contributions to CO_2_ emission reduction as some of the vehicles involved are Battery Electric Vehicles (BEV). Meanwhile, it offers an opportunity to introduce BEV to a broader consumer base. Chen and Kockelman [4] found that, in North America, approximately 51% of carsharing members’ average individual transportation energy use and CO_2_ emissions could be reduced. Secondly, as a beneficial complement to the urban public transport, carsharing mode can provide users with more flexible and comfortable service. By using carsharing, people can arrive at their destination without transfer in the urban public transport system. Thirdly, using carsharing is less expensive for enjoying a high-quality trip, while users can gain the benefits of a private car without paying the cost of ownership. Fourthly, by introducing carsharing, the culture of shared responsibility for the urban transport system can be promoted; shared use mobility initiatives have proven to be powerful mechanisms for reforming the image of the city to a greener and more human-friendly one [5,6].

Carsharing originated in Europe between the 1940s and 1980s, and became popular in the early 1990s. For nearly 20 years, the knowledge and advanced operation technologies of carsharing have spread in the whole world [7]. Specially, as of October 2014, carsharing operated in more than 1531 cities and 33 countries, with approximately 4.8 million carsharing members and 104,000 vehicles [8]. Exploring and understanding of individuals’ acceptance to carsharing is the basic support for making carsharing development planning, policies and strategies. Comprehensive studies have been carried in many developed counties (USA, Australia, Switzerland, Germany, Korea, etc.) on the issue of improving individuals’ willingness to postpone private car purchases and participate in carsharing [9,10,11,12,13,14,15].

However, previous findings in developed countries significantly underrepresent the carsharing development prospect in China. The stage of private car development in China is quite different from that in development countries [16]. In the past decades, with the urbanization development, China has been in a period of rapid urban motorization. As one of the world’s largest automobile markets, China has seen a sharp growth in private vehicles. From 2000 to 2015, private vehicle ownership increased from six million to 141 million, along with the density from five cars per thousand people to 10 cars per thousand people. Additionally, the increase of private vehicles has led to many negative consequences for both the natural environment and the urban development in China, such as energy shortage, climate change, waste, and pollution; congestion, noise and overloaded parking lots; and, finally, urban life redefinition and urban sprawl. Inversely, the vehicle fleet in developed countries increases steadily at a low speed, or even diminishes in size. For example, in 2009, the number of private cars of America decreased, as 14 million cars were scrapped and only 10 million new cars were sold [17]. Moreover, low-quality transport service and inadequate supply of urban public transport have caused urban mobility in China to be increasingly reliant on private cars. However, compared to developed countries, China has obvious disadvantages in terms of road facility density and urban public transport service level. It might be more difficult for Chinese people to postpone private car purchases due to the continuing growth of motorization and car dependency. Thus, exploration of individuals’ acceptance to carsharing mode can reveal fundamental and valuable information about the potential of private car reduction and carsharing promotion, which is quite important to develop policies and operation technologies suitable for China.

As a broad concept, carsharing covers several operational models, including round-trip, one-way and free-floating. Specifically, round-trip carsharing system allows users to rent a vehicle from any station in the system for a short period of time, but must return it back to the original pick up location [9]. One-way carsharing system allows users to rent a vehicle from any station in the system for a short period of time, and return it back to the original station or any other station [18]. Free-floating carsharing system allows users to rent a vehicle at any point in the system, given that there is an available vehicle, and return it back to any point, with an available parking space [19,20]. Since 2010, many pilot cities in China have introduced, or are planning to introduce, carsharing. Some carsharing companies and organizations, such as City Car Club (CCC), Yi Duo, and Germany Car2go Company are developing at an amazing speed. Table 1 lists some basic information about carsharing development in China. However, individuals’ perception on carsharing is still unknown in China. Therefore, empirical studies focusing on the potential factors influencing individuals’ acceptance to carsharing are urgently needed in China to provide better guidance on future practice. It should be noted that, in this study, we focus on the free-floating carsharing system and the vehicles involved are BEVs. Opposed to round-trip and one-way carsharing, there are no fixed stations in the free-floating carsharing system, which allows users to start and end a vehicle rental at any point within a specified area.

This study aims to explore individuals’ acceptance to carsharing including the following three aspects: individuals’ carsharing mode choice behavior under given trip purposes and distances, which is critical for planners to reasonably distribute carsharing infrastructures and vehicles; individuals’ acceptable price to use carsharing, which is vital for operators to accurately fix the price; and individuals’ willingness to participate in carsharing and postpone buying new cars, which is the basic information for environmental protection departments to estimate the contribution of carsharing to the CO_2_ emission reduction. To obtain the required data, a web-based survey was conducted. Moreover, the method of Hierarchical Tree-based Regression (HTBR) was used to characterize the differences in individuals’ acceptance to carsharing between different groups based on individual and external factors. Given the above, this study would contribute to a better understanding of carsharing development direction in China, and make preliminary recommendations for carsharing planning and operation so as to improve its acceptance.

## 2. Literature Review

Carsharing has been a research hotspot for the past two decades. Previous studies have identified numerous factors that influence individuals’ acceptance to carsharing mode. These factors can be generally divided into two main categories: individual-related factors and service-related factors.

Studies related to individual-related factors have been conducted to investigate demographics characteristics of carsharing members, including the gender [21,22], age [12,15,22,23,24], education level [23,25], income [12,23,24,25], children in the household [26,27,28], car ownership [24,25,29], etc. Research on the demographics of carsharing members always point to fairly similar results that individuals with high education level, moderate income or that do not have a car might be more likely to participant in carsharing; and membership of carsharing showed a significant impact on private car ownership reduction.

Specifically, Cousins [21] found that female occupied a higher proportion than male among the sample of people participating in carsharing in UK. Based on the booking data of two carsharing systems in Munich, short-term and long-term predictions on carsharing usage were carried out, and the results indicated that the age structure of a city had distinct influences on the success of carsharing [15]. Cervero [24] conducted a survey of City CarShare, a carsharing organization in San Francisco, and he found that the members were relatively young while 67% of them were between age 20 and 40. Additionally, the members generally had moderate incomes, and approximately 75% of them were from zero-car households. Fairly similar results were obtained by Martin and Shaheen [25]. Through analyzing the data from several North American carsharing organizations, they found that 84% of carsharing members were well-educated, with at least a bachelor’s degree, and 43% of them had incomes less than $60,000. Moreover, the results revealed that 62% of members were zero-vehicle households before joining carsharing. A web-based survey was conducted among participants from electric vehicle sharing programs in Seoul [12]. Results showed that age and income significantly influenced the sharing behaviors. Specifically, participants with higher income were found to be less likely to change their sharing behaviors. Contrary to the findings in previous studies that young people were more likely to participant in carsharing, they found that older people showed higher willingness to continuously participate.

Moreover, extensive studies have proven that carsharing membership has an incentive effect on individuals’ willingness to give up private car ownership. Specially, eighteen months after the Short-Term Auto Rental (STAR) program was established in San Francisco, Walb and Loudon [30] found that 17% of the members sold their cars, and 43% of them would be willing to forgo a car purchase. Katzev [29] studied the early adopters of Car Sharing Portland (CSP), and found that after joining CSP, 26% of them sold their personal cars and 53% given up car purchases. Considering most of previous studies focused on a specific carsharing organization, Transit Cooperative Research Program (TCRP) summarized 31 former studies about carsharing, and pointed out that 21% of respondents would give up cars and 34% reported forgoing car purchases [3]. In view of the rapid spread of carsharing in the world, Shaeen and Cohen [7] made a systematic comparison of carsharing between Europe and North America based on 44 previous studies. The results revealed that each carsharing vehicle could contribute to the reduction of 4–10 private cars in Europe, and 6–23 cars in North America. Moreover, among those joining carsharing, the proportions of participants selling private cars were 15.6–34% in Europe, and 11–29% in North America. The above findings suggest that carsharing has a significant impact on reducing the number of private cars. Clewlow [10] compared the individuals’ attitudes to private car ownership not only between members and non-members, but also between urban and suburban areas in San Francisco. The results suggested that urban carsharing members owned fewer cars than others, and they were more likely to choose environment friendly vehicles.

Except for individual-related factors, previous studies also found that service-related factors significantly influenced the individuals’ willingness to participant in carsharing, such as the convenience of carsharing service [14,29,31], price [11,22,31], types of carsharing vehicles [32,33], etc. The results of an investigation conducted in Europe showed that residents would not like to use carsharing if organizations provided poor services, bad vehicles, inconvenient ways to rental spots, and fixed a high price [14]. Generally, when carsharing service can be obtained conveniently, individuals are more likely to be involved in carsharing. According to the data from Car Sharing Portland (CSP), Katzev [29] found that the distance to the nearest vehicle station was one of the most important predictors of carsharing usage. Individuals living close to vehicle stations tended to have higher use frequency. Based on the data from Car2go services in Austin, Texas, Khan et al. [31] found that land-use level, carsharing parking policy, and number of transit stations had an obvious effect on the usage of free-floating carsharing vehicles. A logistic regression model was applied to identify the impact, and a duration model was applied to quantify the positive correlation with the number of transit stations and parking costs on the likelihood of vehicle rental [31]. Some scholars found that types of carsharing vehicles significantly influenced individuals’ carsharing participant willingness. By conducting an online survey among Car2go users in Germany, Firnkorn and Müller [32] found that compared with gasoline vehicles, individuals were more inclined to forgo a private car purchase when providing BEV in carsharing system. Additionally, previous experiences of driving electric vehicles increased the propensity of users to dispose of their own cars and switch to carsharing. Similar results were founded by Cartenì et al. [22], Wappelhorst et al. [34] and Kim et al. [13].

Based on the above literature review, we can see that a growing body of studies has been carried out worldwide considering a range of carsharing issues. However, in China, the lack of practical operation data leads to relatively less research on carsharing. Representative studies include Shaheen and Martin [35,36], Wang et al. [23], and Hui et al. [37]. Shaheen and Martin [35] implemented a carsharing familiarity and potential demand survey in Beijing, and found that 25% of respondents expressed a high level of interest in carsharing. Another exploratory study was also conducted by Shaheen and Martin [36] to assess the potential for carsharing systems in Beijing, China. The results indicated that integrating carsharing into existing transit networks could become an important mobility option. To evaluate the potential of carsharing in China, Wang et al. [23] compared core distinctions of the taxi systems’ size and competitiveness between key carsharing cities worldwide and Shanghai. They found that carsharing might indeed compete with taxis. Based on the vehicle GPS data of a round-trip carsharing system in Hangzhou, Hui et al. [37] explored the users’ trip chain characteristics.

Clearly, research on carsharing in China is quite limited, not only in quantity, but also in research scope. There are rare relevant studies focusing on individuals’ acceptance to carsharing in China, and most of them generally focused on one city. Additionally, factors associated with individuals’ carsharing participant behaviors and willingness to forgo car purchases in China have never been explored. What makes things more complicated is that due to different development backgrounds of carsharing, some specific factors that are quite important in China have never been analyzed in previous studies. For example, to avoid dramatic increase in private car ownerships, many cities in China, such as Beijing, Shanghai, Shenzhen, etc., have carried out car purchase restriction policies (CPRP). License-plate lottery and auction are two main measures. In cities with CPRP, individuals may need to be lucky enough to win the lottery or pay expensive fees to win the auction. Thus, it is quite difficult for individuals to own private cars in these cities, which brings carsharing development great opportunities. Thus, this study conducts a survey taking various potential influencing factors into consideration, develops hierarchical tree-based regression models to estimate individuals’ acceptance to carsharing, explores the relationships between variables, and, finally, provides practical strategies for establishing and promoting carsharing system in China.

## 3. Method

### 3.1. Survey and Data

To collect the information about influencing factors on individuals’ acceptance of carsharing in China, a questionnaire-based survey was conducted on a professional online survey platform, named as “Questionnaire Star” (http://www.sojump.com/). The web-based survey has great advantages, as it is convenient for respondents to complete the questionnaire without the restrictions of time and geographical areas. Additionally, a well-designed framework of a survey website may help respondents fully understand the purpose of the survey and answer questions properly. More importantly, it minimizes the occurrences of missing answers by advising respondents to check all questions. The survey was conducted online for a length of six months from June 2015 to November 2015. Finally, of the 845 random participants completing the survey, there were 826 valid responses that could be used to analyze participants’ acceptance to carsharing in China, after filtering the incomplete, incorrect and inaccurate data.

In the survey, participants were asked about their: (a) demographic information, such as gender, age, profession, education level, personal income, children in the household and vehicle ownership; (b) awareness of carsharing and lowest acceptable price of carsharing vehicles; (c) living cities’ car-purchase restriction policy (CPRP) and urban public transport service level (UPTSL); (d) carsharing mode choice patterns under different trip purposes and trip distances; (e) highest acceptable price to use carsharing; and (f) willingness to forgo a car purchase and participating in carsharing. It should be noted that the trip purpose was divided into six categories: commute, shopping, going to the doctor, visiting relatives and friends, business activities and ferrying children. The trip distance was also divided into six intervals: <3 km, 3–10 km, 10–20 km, 20–30 km, 30–40 km, and >40 km. Each participant had to make twelve repeated choice decisions under different trip purposes and trip distances while other explanatory variables related to the participant remain the same. Thus, trip purpose and trip distance in this study could be regarded as repeated variables.

Table 2 shows the relevant statistical information about valid collected responses. Participants’ education level was divided into three different categories according to their highest school record: low-education participants who had never been to a university; middle-education participants who were studying for or had obtained a bachelor’s degree; and high-education participants who were studying for or had obtained a post-graduate degree. Similarly, personal income level was also divided into three categories: low-income participants with less than 5000 Yuan per month; middle-income participants with income between 5000 and 10,000 Yuan per month; and high-income participants with more than 10,000 Yuan per month. The exchange rate between U.S. dollars and RMB is about 6.9, i.e., 1 USD is equal to 6.9 Yuan.

### 3.2. Hierarchical Tree-Based Regression (HTBR) Method

Hierarchical Tree-based Regression (HTBR) is a flexible non-parametric statistical method for dealing with prediction and classification problems [38] that has been widely applied for research in travel behavior in recent years. Similar to forward stepwise regression, HTBR can be regarded as a forward step-wise variable selection method [39]. HTBR classifies observations by recursively partitioning the predictor space [40]. In the process of iterative partitioning, a tree structure is generated by dividing the dataset into subgroups [41]. Through numerical search procedures, HTBR iteratively asks and answers the following two questions: whether variables offered in the model should be selected to produce the maximum reduction in variability of the response; and the threshold values of all the selected variables [39]. The above procedure continues with each subgroup being further subdivided until a desirable end condition is met: the node size of the last split has met minimum population criteria or minimum deviance criteria at a node have been met.

In tree-structured representations, there are three kinds of nodes, the root node which represents the entire dataset and is at the top of the tree; the terminal node, which is not to be split any further and is at the bottom of the tree; and the internal node, which is between the root node and the terminal node. When constructing a tree, the root node and the internal nodes are split sequentially applying a set of decision rules. Each decision rule is used to form branches connecting the root node to the terminal nodes at a certain level of the tree [40]. Moreover, when a node is split, it can be called the parent node, while its splitting node can be called the child node. The tree grows as a result of the testing of partitioning data at the parent nodes. The outgoing branches, i.e., child nodes, of a parent node correspond to all the possible outcomes of the test at the node [42].

Compared with parametric statistical methods, HTBR has several advantages. Firstly, most parametric statistical methods need to propose model assumptions and pre-define potential relationships between dependent and independent variables, i.e., between the target and the predictors, whereas HTBR does not require variables being selected in advance or a specified assumption of function form [43,44]. Thus, HTBR can effectively avoid unreasonable assumptions that may lead to erroneous estimation [45]. Secondly, by using automated selection methods such as stepwise selection techniques and best subsets algorithms, HTBR can straightforwardly yield predictions for dependent variables and identify subgroups of target variable following the optimal splitting rules [44,46]. Thirdly, for regression analysis methods, the variable selection might be an issue when involving many possible independent variables. However, HTBR can effectively deals with large datasets containing a large number of independent variables, and obtain significant results by selecting only a few important independent variables [47]. Fourthly, datasets with complex structure can also be effectively handled by HTBR, such as non-homogeneity and multi-collinearity between independent variables. In addition, both continuous variables and nominal variables can be handled. Finally, the visually graphical representation of the model can make the results more easily understood across disciplines, and also by non-statisticians [47].

In this study, there were three dependent variables, including participants’ carsharing mode choice, highest acceptable price to use carsharing, and willingness to forgo car purchases. Moreover, independent variables were multi-categorical nominal variables. Therefore, HTBR method is quite suited for exploring the influencing relationships between independent and dependent variables. The HTBR analyses in this study were carried out using the IBM SPSS 22.0 software package (IBM, North Castle, NY, USA). The specifications for tree construction include: CHAID algorithm was applied; the maximum tree depth was set as 3 levels; and the significance values for splitting nodes and merging categories were set as 0.05. The minimum number of cases for parent nodes was set as 100 and the minimum number of cases for child nodes was set as 50.

## 4. Descriptive Analysis Findings

### 4.1. Carsharing Mode Choice under Different Trip Purposes and Trip Distances

**Figure 1:**
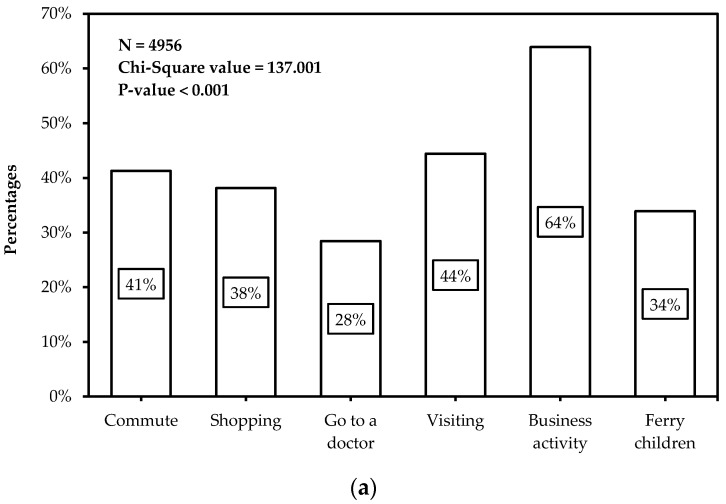

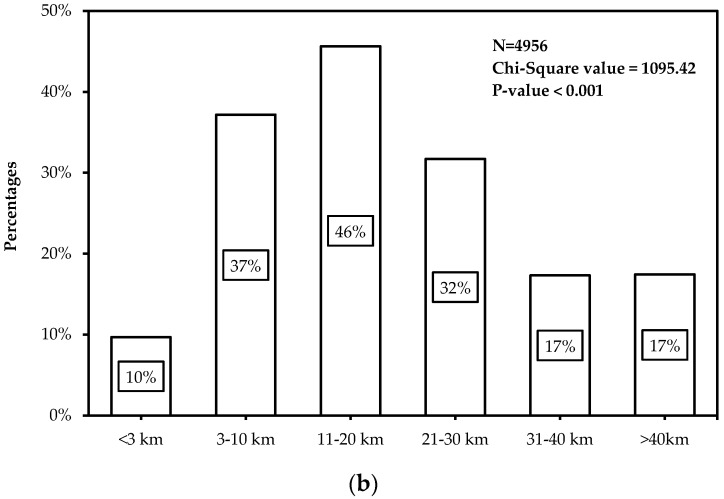
Participants’ carsharing mode choices under: (**a**) different trip purposes; and (**b**) different trip distances.

Figure 1 shows the distribution of participants’ carsharing mode choices under different trip purposes and different trip distances. In Figure 1a, it could be found that, among the six trip purposes, business activities occupied the highest choosing proportion of carsharing mode, followed by visiting friends and relatives, commuting, shopping, drop-off and pick-up of children, and finally go to the doctor. Specifically, participants’ choosing rate ranges from a minimum of 28.5% to a maximum of 64%. In Figure 1b, 377 participants showed willingness to use carsharing mode when the trip distance was 11–20 km, accounting for the highest proportion, 46%. The results revealed that other relatively competitive trip distance intervals of carsharing mode included 3–10 km and 21–30 km, the choice rate of which were both higher than 30%. When the trip distance was within the other three intervals, <3 km, 31–40 km and >40 km, carsharing mode was not attractive to participants.

### 4.2. Highest Acceptable Price for Using Carsharing

**Figure 2:**
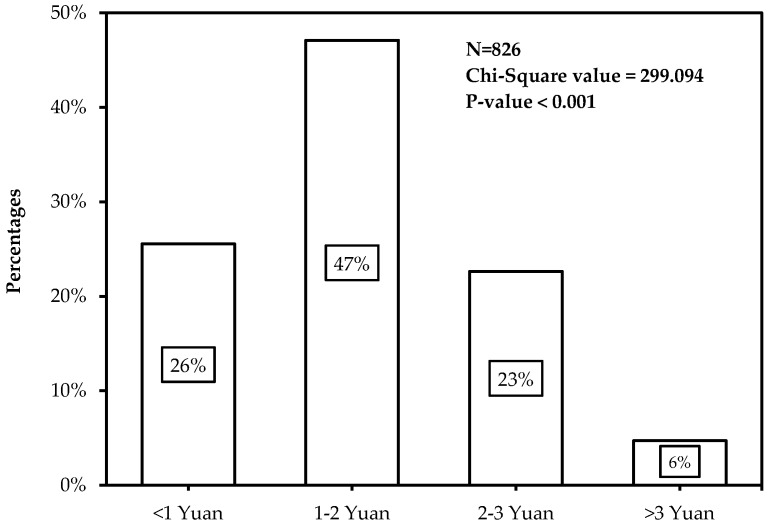
Participants’ highest acceptable price to use carsharing.

Figure 2 displays the distribution of participants’ highest acceptable price to use carsharing. It should be stated here that the cost in this paper was calculated by minutes. Results obviously revealed that most of participants (47.1%) could accept the price between 1 and 2 Yuan. In total, 25.5% of participants insisted that only when the price was less than 1 Yuan they would consider using carsharing; 22.6% of participants could accept the price between 2 and 3 Yuan; and only 39 participants (4.7%) would be willing to pay more than 3 Yuan.

Table 2 lists the statistical information of participants’ highest acceptable price based on different categories of independent variables. In Table 3, the following general characteristics of participants’ acceptable price could be identified.

- Except CPRP, UPTSL, participants’ age, education level and children in the household, all the other variables showed significant effects on the highest acceptable price.
- Compared with females, more males could accept relatively higher trip cost. Specifically, when the price was more than 2 Yuan, 32% of males could accept, but only 23% females could accept.
- Thirty-one percent of non-office workers thought that the price should be smaller than 1 Yuan, while the percentage of office workers (23%) was a little lower. Inversely, 34% of office workers showed acceptance of the price higher than 2 Yuan, while that of non-office workers was 22%. The results revealed that office workers were willing to pay more to use carsharing than non-office workers.
- With the increase of income, the percentage of accepting high price (>2 Yuan) would also increase: 18% for low-income, 33% for middle-income, and 48% for high-income.
- Participants who have private cars were found to be more willing to accept high price than those not having cars. Particularly, the accepting percentage of each price interval for participants who have private cars was 48% for 1–2 Yuan, 27% for 2–3 Yuan, and 5% for >3 Yuan, which were all higher than percentages of participants not having a private.
- Results showed that participants who know about carsharing could accept higher price. Specifically, 30% of participants who know about carsharing could accept the price between 2 and 3 Yuan, which was 16% higher than those who do not know about carsharing.
- When price of carsharing vehicles is less than 100,000, only 14% of them were willing to pay more than 2 Yuan. However, with the price of carsharing vehicles increasing to 200,000, more than 50% of participants could accept a price over 2 Yuan. It indicated that participants would be willing to pay more when carsharing vehicles are more expensive.

### 4.3. Willingness to Forgo Car Purchases

**Figure 3:**
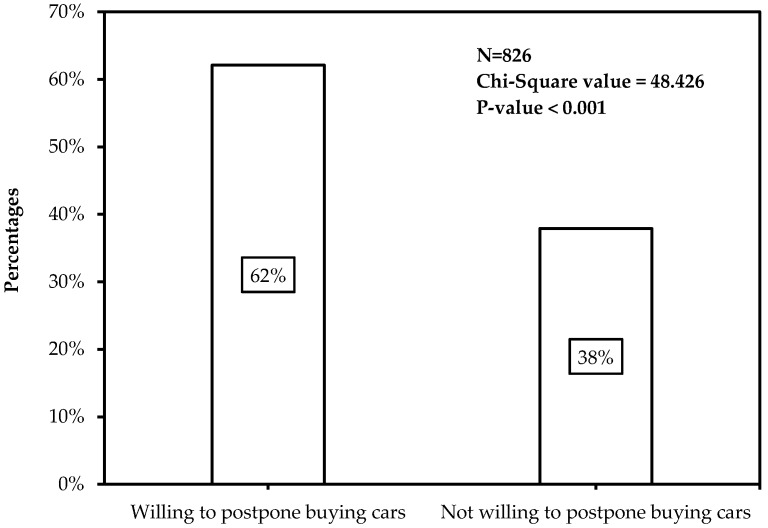
Participants’ willingness to forgo car purchases.

To measure the influence of carsharing on the private car reduction, participants were asked whether they were willing to give up buying new cars if carsharing service could satisfy their travel demand. Figure 3 shows the distribution of participants’ willingness to forgo car purchases. It was obvious that more participants (62.1%) would like to quit buying a new car.

Table 4 displays the statistical information of participants’ willingness to forgo car purchases based on different categories of independent variables. In Table 4, we could identify the following general characteristics:

- Variables, including CPRP, UPTSL, gender, income level, car ownership, awareness of carsharing, and price of carsharing vehicles, showed significant impacts on participants’ willingness to forgo car purchases and participant in carsharing.
- When living in a city without CPRP, 55% of participants were inclined to give up buying a new car. The proportion would increase to 66% among participants living in a city with CPRP. The results revealed that CPRP could provide a meaningful pathway to reduce private car ownership and promote carsharing.
- Only 5% of participants with low satisfaction on UPTSL would be willing to use carsharing instead of buying a car. Conversely, when having high satisfaction, more participants (37%) showed interests in forgoing car purchases. It indicated that with the increase of participants’ satisfaction on UPTSL, their willingness to forgo buying cars would dramatically increase.
- Results showed that males were more insistent on buying cars. The proportion of male participants accepting to forgoing car purchases was 59%, while that of females was 65%.
- A clear relationship between participants’ willingness to give up buying cars and their income level was that the former increased with the latter. Particularly, the willing proportion of participants from the low-income group was 58%, while that of participants from the high-income group was 68%.
- Seventy-five percent of participants who have cars were willing to forgo car purchases, which was obviously more than those who do not have cars (44%). It indicated that carsharing had a positive impact on reducing the number of cars in the household.
- Participants who know about carsharing would be more likely to accept forgoing car purchases (75%) than those who do not know about carsharing (48%). Thus, carsharing organizations should pay more attention to widely advertising carsharing.

## 5. HTBR Modeling Results

Four HTBR models were constructed in this study. It should be noted that Model #1 and Model #2 were, respectively, constructed for predicting participants’ carsharing mode choice under different trip purposes and trip distances. For data inputs, the four models had the same nine prediction variables (independent variables): CPRP, UPTSL, participants’ gender, profession, income, children in the household, car ownership, awareness of carsharing, and lowest acceptable price of carsharing vehicles. In addition, the trip purpose and trip distance were regarded as two classification variables being put into HTBR Model #1 and Model #2, respectively. 

### 5.1. HTBR Model #1 and Model #2–Predicting Participants’ Carsharing Mode Choice

**Figure 4:**
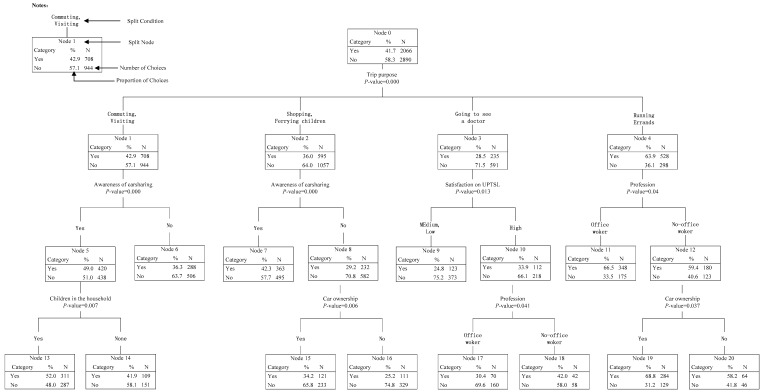
Hierarchical Tree-based Regression (HTBR) Model #1: Predicting predicting participants’ carsharing mode choice under different purposes.

**Figure 5:**
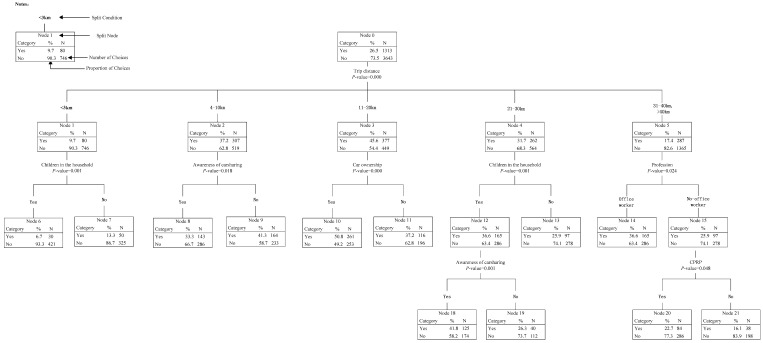
HTBR Model #2: Predicting predicting participants’ carsharing mode choice under different distances.

The results of Model #1 and Model #2 are, respectively, displayed in Figure 4 and Figure 5. The final tree structures for participants’ carsharing mode choice under different trip purposes and different trip distances, respectively, involved six splitting variables (trip purpose, awareness of carsharing, UPTSL, profession, children in household, and car ownership), and six splitting variables (trip distance, children in household, awareness of carsharing, car ownership, profession, and CPRP). 

As shown in Figure 4, the tree could be classified as follows. In the first level of the tree, the participants’ carsharing mode choice behavior (Node 0) was divided into four child nodes (Node 1–4) according to different trip purposes. In the second level, Node 1 and Node 2 were both divided into two child nodes (Node 5–8) by participants’ awareness of carsharing; Node 3 was divided into two child nodes (Node 9–10) by participants’ satisfaction on UPTSL; and Node 4 was divided into two child nodes (Node 11–12) by participants’ profession. In the third level, Node 5 was divided into two child nodes (Node 13–14) by children in the household; Node 8 and Node 12 were both divided into two child nodes (Node 15–16 and Node 19–20, respectively) by car ownership; and Node 10 was divided into two child nodes (Node 17–18) by participants’ profession. The detailed characteristics could be identified as follows.

*In the first level*: The purposes of commuting and visiting were classified into the same subgroup, while shopping and ferrying children were in the same subgroup. The results meant that participants tended to make the same choice of whether to use carsharing mode under purposes in the same subgroups. Moreover, the proportion of participants choosing carsharing mode for business activities was the highest (63.9%), followed by commuting and visiting (42.9%), shopping and ferrying children (36.0%), and finally going to the doctor (28.5%).

*In the second level:* When commuting, visiting, shopping or ferrying children, participants’ awareness of carsharing was the most important influencing factor for carsharing mode choice, while participants’ satisfaction on UPTSL was the most important when going to the doctor, and profession was the most important when travelling for business activities. From Node 5 to Note 8, it could be found that participants who know about carsharing were more likely to use it compared with those who do not know about carsharing. When going to the doctor, participants with high satisfaction on UPTSL would be more willing to use carsharing. When travelling for business activities, 66.5% of office workers chose to use carsharing, which was 7.1% higher than the choosing proportion of non-office workers.

*In the third level:* When commuting and visiting, children in the household significantly influenced the choice behavior of participants who know about carsharing: participants who have children were more likely to use carsharing; when shopping and ferrying children, the choice behavior of participants who do not know about carsharing was influenced by the car ownership: participants who have cars were more likely to use carsharing. The same conclusion could be addressed when analyzing the choice behavior of office workers when travelling for business activities; when going to the doctor, participants’ profession was the key factor influencing the choice behavior of participants with high satisfaction on UPTSL: the proportion of non-office workers choosing carsharing (42.0%) was much higher than that of office workers (30.4%).

As shown in Figure 5, the tree could be classified as follows. In the first level of the tree, the participants’ carsharing mode choice (Node 0) was divided into five child nodes (Node 1–5) according to different trip distances. In the second level, Node 1 and Node 4 were both divided into two child nodes (Node 6–7 and Node 12–13) by children in the household; Node 2 was divided into two child nodes (Node 8–9) by participants’ awareness of carsharing; Node 3 was divided into two child nodes (Node 10–11) by car ownership; and Node 5 was divided into two child nodes (Node 14–15) by participants’ profession. In the third level, Node 9 and Node 15 were both divided into two child nodes (Node 16–17 and Node 20–21) by CPRP; and Node 12 was divided into two child nodes (Node 18–19) by participants’ awareness of carsharing. The detailed characteristics could be identified as follows:

*In the first level*: The distance intervals of 31–40 km and >40 km were classified into the same subgroup, which meant that participants’ choice behavior characteristics was similar when the trip distance was over 30 km. Moreover, the results revealed that participants were not willing to use carsharing mode when their trip distance was shorter than 3 km or longer than 30 km.

*In the second level:* For trip distance <3 km and 21–30 km, children in the household was the most important classification factor for participants’ choice, while participants’ awareness of carsharing mode, car ownership and profession were the most important for trip distance 4–10 km, 11–20 km and >30 km, respectively. Specifically, for trip distance <3 km, participants not having children were more likely to use carsharing. Inversely, when the trip distance increased to 21–30 km, 36.6% of participants who have children were willing to use carsharing, which was 10.7% higher than those not having children. For trip distance 4–10 km, it was found that some participants would still not willing to use carsharing even though they were aware of it. For trip distance 11–20 km, 49.2% of participants who have cars accepted to use carsharing, which was much higher than participants not having cars (37.2%). For trip distance >30 km, 36.6% of office workers chose to use carsharing, which was 10.7% higher than that of non-office workers.

*In the third level:* When trip distance was between 21 km and 30 km, the choice behavior of participants who have children was significantly influenced by their awareness of carsharing: 41.8% of participants who know about carsharing chose to use the mode while only 26.3% of participants who do not know about carsharing were willing to use. When trip distance was over 30 km, CPRP had an obvious impact on the choice behavior of non-office workers: participants living in a city having CPRP were more likely to use carsharing.

### 5.2. HTBR Model #3: Predicting Participants’ Highest Acceptable Price for Using Carsharing

**Figure 6:**
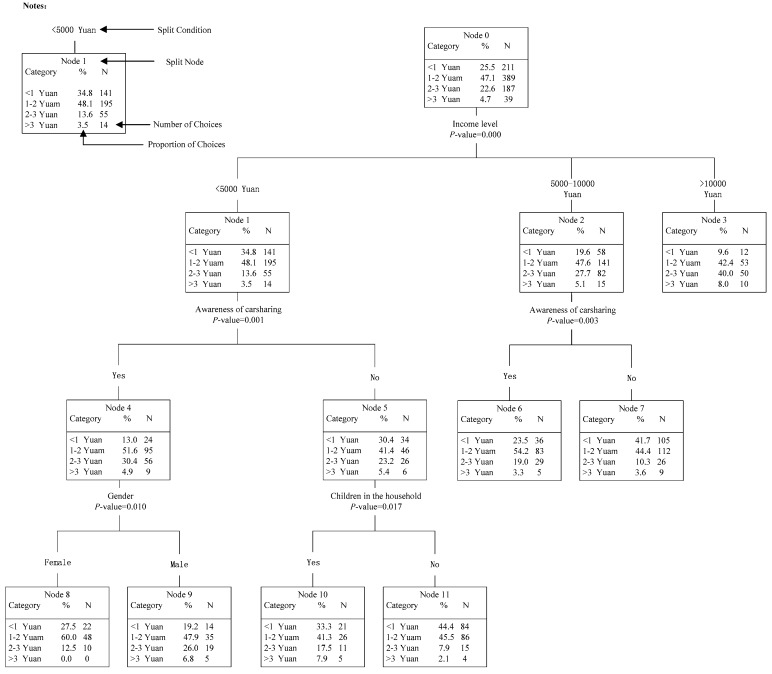
HTBR Model #3: Predicting participants’ highest acceptable price to use carsharing.

The results of Model #3, displayed in Figure 6, are applied for predicting participants’ highest acceptable price. The final tree structure involved four splitting variables, including participants’ income level, awareness of carsharing, gender and children in the household. The results meant that the above four variables significantly influenced participants’ highest acceptable price to use carsharing service, among which income level was the most important factor, followed by awareness of carsharing, gender and finally children in the household.

As shown in Figure 6, the tree could be classified as follows. In the first level of the tree, the participants’ highest acceptable price (Node 0) was divided into three child nodes (Node 1–3) according to participants’ income level. In the second level, Node 1 and Node 2 were both divided into two child nodes (Node 4–5 and Node 6–7, respectively) by participants’ awareness of carsharing. In the third level, Node 4 was divided into two child nodes (Node 8–9) by gender; and Node 5 was divided into two child nodes (Node 10–11) by children in the household. The detailed characteristics could be identified as follows:

*In the first level*: It was found that participants’ income level was the most important influencing factor on participants’ highest acceptable price. For income <5000 Yuan, 34.8% of participants could only accept the price lower than 1 Yuan, which was much higher than proportions of participants with income 5000–10,000 Yuan (19.6%) and >10,000 Yuan (9.6%). Similar trend could also be found for income between 1 Yuan and 2 Yuan. Inversely, for income >10,000, 48% of participants could accept the price over 2 Yuan, which was, respectively, 15.2% and 30.9% higher than the proportions of participants with income <5000 Yuan and 5000–10,000 Yuan. The results revealed that with the increase of income, participants would be willing to pay more to use carsharing.

*In the second level:* For income <10,000, participants’ awareness of carsharing was the most important classification factor for participants’ highest acceptable price. Specifically, from Node 4 to Node 7, it could be found that participants who know about carsharing were more likely to pay higher price to use carsharing than those never hearing about carsharing.

*In the third level:* For income <5000 Yuan, the highest acceptable price of participants who know about carsharing was significantly influenced by gender: 32.8% of males could accept the price over 2 Yuan, which was much higher than the proportion of females (12.5%). For income 5000–10,000 Yuan, when participants who do not know about carsharing, whether they have children had an obvious impact on participants’ highest acceptable price: if having children, participants tended to accept higher price.

### 5.3. HTBR Model #4: Predicting Participants’ Willingness to Forgo Car Purchases

**Figure 7:**
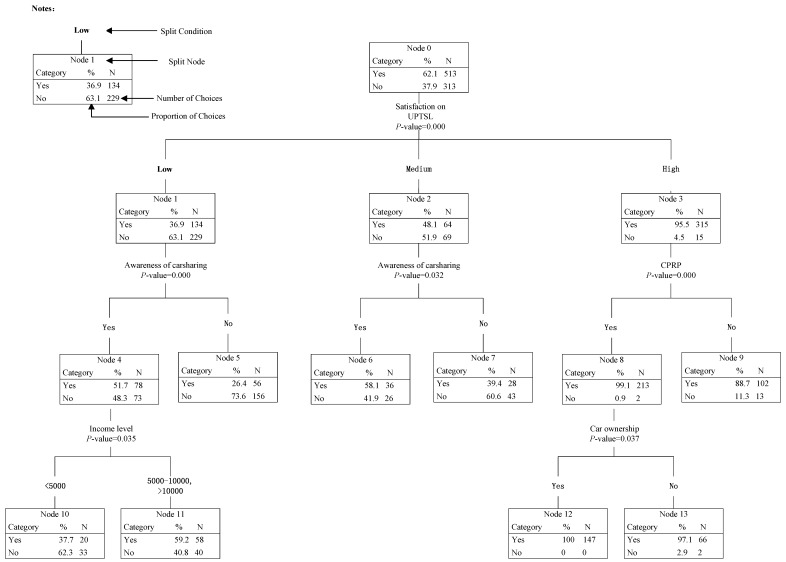
HTBR Model #4: Predicting participants’ willingness to forgo car purchases.

The results of Model #4 are shown in Figure 7. The final tree structure involved five splitting variables: participants’ satisfaction on URTSL, awareness of carsharing, CPRP, income level and car ownership. The results indicated that the above five variables significantly influenced participants’ willingness to forgo car purchases, among which satisfaction on URTSL was the most important factor.

As shown in Figure 7, the tree could be classified as follows. In the first level of the tree, the participants’ willingness to forgo car purchases (Node 0) was divided into three child nodes (Node 1–3) according to participants’ satisfaction on URTSL. In the second level, Node 1 and Node 2 were both divided into two child nodes (Node 4–5 and Node 6–7, respectively) by participants’ awareness of carsharing; and Node 3 was divided into two child nodes (Node 8–9) by CPRP. In the third level, Node 4 was divided into two child nodes (Node 8–9) by income level; and Node 8 was divided into two child nodes (Node 10–11) by car ownership. The detailed characteristics could be identified as follows:

*In the first level*: It was found that participants’ satisfaction on UPTSL was the most important influencing factor on participants’ willingness to give up buying new cars. Totally, 95.5% of participants with high satisfaction considered giving up car purchases if carsharing could satisfy their travel demand, while the proportions of participants with medium and low satisfaction were, respectively, 48.1% and 36.9%. It indicated that the higher the UPTSL was, the less new private cars would be by introducing carsharing.

*In the second level:* participants’ awareness of carsharing were found to be the most important influencing factor on the willingness to forgo buying new cars of participants with low or medium satisfaction on UPTSL. From Node 4 to Node 7, it was obvious that participants who know about carsharing were much more willing to forgo buying new cars. CPRP was found to be the key factor influencing the willingness to forgo buying new cars of participants with high satisfaction. When living in a city having CPRP, 99.1% of participants were willing to forgo buying new cars, which was much higher than the proportion of participants living in a city not having CPRP.

*In the third level:* For participants with low satisfaction on UPTSL and who know about carsharing, income was the most important influencing factor: when income was less than 5000 Yuan, 37.7% of participants were willing to forgo buying new cars, which was much lower than that of participants with more than 5000 Yuan income (59.2%). When living in a city having CPRP, car ownership had an obvious impact on the willingness to forgo buying new cars of participants with high satisfaction on UPTSL: if having cars, all participants were willing to forgo buying a new car.

## 6. Conclusions

Using the data collected from an online survey, this study has identified factors that influence individuals’ acceptance to carsharing in China, including individuals’ carsharing mode choice behavior under given trip purposes and distances, highest acceptable price to use carsharing, and willingness to forgo car purchases. Through correlation description analyses and HTBR methods, some important findings have been obtained. Detailed relationships between factors and individuals’ acceptance to carsharing can provide better insights into the development potential of carsharing in China. Moreover, the findings in this paper can also contribute to provide basic information for the theory of behavior change in transportation. The results showed that many responders in China were willing to participate in carsharing. As a flexible mobility option, carsharing complements public transportation and slow modes by offering the flexibility of private cars without owning private cars. Thus, carsharing may potentially foster more sustainable mobility by facilitating multi-modal travel behavior. Furthermore, carsharing could reduce private car ownership levels in the longer run.

As with any new concept, carsharing faces challenges in getting a stronghold as a sustainable urban transport mode. Based on findings in this paper, some challenges are outlined and corresponding policies are proposed to provide guidance for improving individuals’ acceptance to carsharing in China, which may also be applicable to other countries. Strategies promoting carsharing development can be proposed from the following four aspects.

### 6.1. Improve Individuals’ Awareness of Carsharing

As expected, individuals’ awareness of carsharing showed distinct impacts on individuals’ acceptance to carsharing. Results indicated that individuals who know about carsharing were more likely to use carsharing, pay higher price and forgo car purchases. Similarly, results were founded by Millard-Ball et al. [3]. Based on the data from Transit Cooperative Research Program (TCRP), they found that only 0.03% of the US urban population joined carsharing in 2004, and the biggest hurdle for the adoption was the lack of awareness and understanding about the concept. Unfortunately, according to our survey, approximately half of participants (48.1%) had never heard about carsharing, which meant that many individuals in China did not understand what carsharing was, how and where it worked, and how it differed from traditional ridesharing. Thus, to popularize carsharing in China and highlight its advantages in private car reduction, it is quite urgent to make great efforts to dispel individuals’ confusion about this new concept and increase their awareness.

Marketing is considered to be the most effective way to promote better understanding of carsharing among the public. Specially, marketing can be carried out by different methods, such as delivering information on websites and in newsletters, distributing materials at transportation fairs, encouraging media coverage, issuing press releases, offering links from transportation department website to carsharing service website, and forming nation-wide organizations to educate policy makers and the wider public as to the role and benefits of carsharing. For example, to spread carsharing concept and make individuals aware of carsharing’s beneficial potential, Zipcar organization carried out some strategies to create awareness for their service in the USA, Canada, Germany, France, United Kingdom, etc., including: setting up a website with extensive information regarding the service; disseminating information to a large group of people by website and magazine advertising; cooperating with some organizations or education centers; and establishing alliances with famous vehicle producers.

### 6.2. Provide Government Supports

The results also showed that variables related to participants’ living city significantly influenced their willingness to participate in carsharing. Particularly, when CPRP was carried out in a city or the UPTSL was high, individuals were more willing to give up buying new cars. Similarly, previous research found that individuals would have less motivation to use carsharing in areas with poor public transportation [48]. Thus, to realize the goals of private car reduction, great efforts should be made to strengthen the urban management policies as well as improve the UPTSL. Measures controlling the increase of private cars, such as CPRP and auto plate auction policy, should continue to be carried out, especially in metropolises such as Beijing, Shanghai, Shenzhen, etc.

Previous studies have also reported that the government could play an important role in fostering carsharing market development, primarily in the form of providing financial support and subsidized parking [49,50,51]. For example, some 80% of carsharing organizations in the USA are financial supported by governments through tax incentives, starting investments, emergency risk fund support, etc. [50]. For the early development stage of carsharing in China, the government can learn from the developed countries to give some financial support to enhance the social and environmental benefits brought by carsharing. Moreover, as another significant measure to foster carsharing development, parking subsidies can be quite significant, particularly in congested areas. With reference of experiences from developed countries, finding parking spaces are the largest barriers to carsharing expansion. In China, both the on-street and off-street parking are controlled by government agencies. Permission of parking spaces for carsharing has been one of the most tangible forms of governments’ supports. Relevant plans for carsharing parking lots should be carried out to improve individuals’ acceptance, such as marking parking zones for carsharing, setting free metered parking on-street, offering discounts in municipal lots, etc.

Additionally, carsharing can be considered in urban and traffic development planning documents as an important sustainable strategy to provide strong support for carsharing development from the government. Besides, planning schemes of carsharing pilot projects in China can be published to guide the implementation of carsharing system, especially for reasonably allocating carsharing service.

### 6.3. Reasonably Allocate Carsharing Service

The results in our paper might be some of significance to give insight into the reasonable allocation of carsharing service, especially for carsharing vehicles and BEV charging spots. In this paper, trip purpose and trip distance were found to be the most two important influencing factors of carsharing mode choice behavior. For trip purpose, results showed that most individuals chose to use carsharing when travelling for business activity, followed by visiting friends and relatives, commuting, shopping, ferrying children, and finally going to the doctor. Generally, when travelling for business activities, individuals travel between office blocks. The other five purposes are between neighborhoods when visiting friends and relatives; neighborhoods and office blocks or schools when commuting and ferrying children; neighborhoods and commercial districts when shopping; and neighborhoods and hospitals when going to the doctor. The results indicate that the demand for carsharing in China mainly distributes in office blocks and neighborhoods. Thus, more carsharing vehicles should be allocated in neighborhoods, major employment centers, and central business areas. Similar results can be found in previous research [12]. As one of the most important factors influencing the battery remaining capacity of BEV, the trip distance should be considered when allocating the BEV charging spots. In this study, it was found that 11–20 km (46%) was the most competitive trip distance interval, followed by 3–10 km (37.2%), 21–30 km (31.7%), >40 km (17.4%), 31–40 km (17.3%), and finally <3 km (9.7%). The results indicated that carsharing was attractive to individuals when their trip distance interval was 3–30 km. The above findings about attractive trip distance interval can provide important basic information for further exploring and quantitatively calculating the optimal layout plan of carsharing infrastructures, such as parking lots, battery-charging stations and so on. 

### 6.4. Provide High-Quality and Diversified Service

Carsharing operators should focus on providing high-quality and diversified service to satisfy individuals’ different demands. According to the findings in this paper, the relatively competitive price range in China was <2 Yuan per minute. Interestingly, in China, the current average price to use carsharing is about 1.5 Yuan per minute. In Germany, the price is a little higher for about 0.25 EURO per minute (nearly 1.85 Yuan) [33]. Moreover, participants’ highest acceptable price to use carsharing was obviously effected by participants’ socio-demographic characteristics. The results showed that males were more likely to pay higher price than females and participants with higher-income could accept higher price. In addition, participants hoping for high-price carsharing vehicle showed more willingness to pay more. Thus, to satisfy the diversified demand, carsharing operators should provide various choices of carsharing vehicles for individuals and reasonably set the price. Vehicles of different price levels can be introduced into carsharing system, and the prices can be set accordingly. Moreover, relatively expensive cars can be allocated around high-class residence communities, since individuals living there always have higher incomes. However, carsharing operators do not need to invest much money to purchase upscale cars, because, in this study, it was found that only 4% of participants hoped that the price of carsharing vehicle is more than 300 thousand Yuan. 

## Figures and Tables

**Table 1:** Basic information about carsharing development in China.

Company	Operation Mode	Rental Time Unit	Battery Electric Vehicle (BEV)	Founded Time (Year)
eHi	One-way	Day	×	2006
Shenzhou	One-way	Day	×	2007
City Car Club	Round-trip	Day	×	2010
YiDuo	One-way	Day	×	2010
GreenGo	Round-trip	Minute	√	2014
Car2go	Free-floating	Minute	×	2015
PanDa	One-way	Minute	√	2015
Hertz	One-way	Day	×	2015
ZhiDou	Free-floating	Minute	√	2015
EVCARD	One-way	Minute	√	2015
TOGO	Free-floating	Minute	×	2015
YouChe	One-way	Minute	√	2015
MoCar	Free-floating	Minute	×	2016
LeShare	One-way	Minute	√	2016
JiaBei	One-way	Minute	√	2016
DaDa	One-way	Minute	√	2016
ShareGo	One-way	Minute	√	2016
GoFun	One-way	Minute	√	2017
eVpop	One-way	Minute	√	2017

**Table 2:** Independent variables used in the study.

Independent Variables	Description/Levels	Summary Statistics	
		N	%
Car purchase restriction policy (CPRP)	Have	546	33.9
	Not have	280	66.1
Urban public transport service level (UPTSL)	Low	363	43.9
	Medium	133	16.1
	High	330	40
Gender	Male	420	50.8
	Female	406	49.2
Age (year)	<20	52	6.3
	21–30	472	57.1
	31–40	233	28.2
	41–50	53	6.4
	Above 50	16	1.9
Profession	Office worker	523	63.3
	Non-office worker	303	36.7
Education level	Low-education	110	13.3
	Middle-education	612	74.1
	High-education	104	12.6
Personal income (¥)	Low-income	405	49.0
	Middle-income	296	34.8
	High-income	125	15.1
Children in the household	None	375	45.4
	Yes	451	54.6
Car ownership	Have a car	514	62.2
	Do not have a car	312	37.8
Awareness of carsharing	Know about carsharing	429	51.9
	Do not know about carsharing	397	48.1
Price of carsharing vehicles	Below 100,000 Yuan	336	40.7
	100 to 200,000 Yuan	319	38.6
	200 to 300,000 Yuan	138	16.7
	Above 300,000 Yuan	33	4.0
Trip purpose	Commute	826	16.7
	Shopping	826	16.7
	Go to the doctor	826	16.7
	Visit relatives and friends	826	16.7
	Business activity	826	16.7
	Ferry children	826	16.7
Trip distance	Trip distance less than 3 km	826	16.7
	Trip distance between 3 and 10 km	826	16.7
	Trip distance between 10 and 20 km	826	16.7
	Trip distance between 20 and 30 km	826	16.7
	Trip distance between 30 and 40 km	826	16.7
	Trip distance more than 40 km	826	16.7

**Table 3:** Statistical information of participants’ highest acceptable price based on different categories of independent variables.

Independent Variables	<1 Yuan		1–2 Yuan		2–3 Yuan		>3 Yuan		Total	
	N	%	N	%	N	%	N	%	N	%
*By CPRP*										
Have	126	23	264	48	133	25	23	4	546	100
Not have	85	30	125	45	54	19	16	6	280	100
*By UPTSL*										
Low	102	28	165	46	79	22	17	5	363	100
Medium	38	29	59	44	28	21	8	6	133	100
High	71	22	165	50	80	24	14	4	330	100
*By gender*										
Male	98	23	189	45	108	26	25	6	420	100
Female	113	28	200	50	79	20	14	3	406	100
*By age (year*)										
<20	15	29	22	42	11	21	4	8	52	100
21–30	121	26	233	49	99	21	19	4	472	100
31–40	51	23	106	48	64	29	12	5	233	100
41–50	16	30	23	43	12	23	2	4	53	100
>50	8	50	5	31	1	6	2	13	16	100
*By profession*										
Non-office worker	93	31	142	47	57	19	11	3	303	100
Office worker	118	23	247	47	130	25	28	5	523	100
*By monthly income*										
Low-income	141	35	195	48	55	14	14	4	405	100
Middle-income	58	20	141	48	82	28	15	5	296	100
High-income	12	10	53	42	50	40	10	8	125	100
*By education level*										
Low-education	35	32	53	48	15	14	7	6	110	100
Middle-education	152	25	283	46	149	24	28	5	612	100
High-education	24	23	53	51	23	22	4	4	104	100
*By child*										
No	116	31	180	48	63	17	16	4	375	100
Yes	95	21	209	46	124	27	23	5	451	100
*By car ownership*										
Have a car	101	20	245	48	141	27	27	5	514	100
Do not have a car	110	35	144	46	46	15	12	4	312	100
*By awareness of carsharing*										
Know about carsharing	67	16	211	49	130	30	21	5	429	100
Do not know about carsharing	144	36	178	45	57	14	18	5	397	100
*By price of carsharing vehicle*										
Below 100,000 Yuan	135	40	154	46	35	10	12	4	336	100
100 to 200,000 Yuan	66	21	166	52	73	23	14	4	319	100
200 to 300,000 Yuan	8	6	57	41	63	46	10	7	138	100
Above 300,000 Yuan	2	6	12	36	16	49	3	9	33	100

**Table 4:** Participants’ willingness to forgo car purchases based on different categories of independent variables.

Independent Variables	Willing to Forgo Car Purchases		Not Willing to Forgo Car Purchases	
	N	%	N	%
*By CPRP*				
Have	359	66	187	34
Not have	154	55	126	45
*By UPTSL*				
Low	134	37	229	63
Medium	64	48	69	52
High	315	95	15	5
*By gender*				
Male	256	61	164	39
Female	257	63	149	37
*By age (year)*				
<20	21	40	31	60
21–30	280	59	192	41
31–40	163	70	70	30
41–50	38	72	15	28
>50	11	69	5	31
*By profession*				
Non-office worker	167	55	136	45
Office worker	346	66	177	34
*By monthly income*				
Low-income	234	58	171	42
Middle-income	194	66	102	34
High-income	85	68	40	32
*By education level*				
Low-education	66	60	44	40
Middle-education	390	64	222	36
High-education	57	55	47	45
*By child*				
No	194	52	181	48
Yes	319	71	132	29
*By car ownership*				
Have a car	340	66	174	34
Do not have a car	173	55	139	45
*By awareness of carsharing*				
Know about carsharing	324	76	105	24
Do not know about carsharing	189	47	208	53
*By price of carsharing vehicle*				
Below 100,000 Yuan	190	56	146	44
100 to 200,000 Yuan	207	65	112	35
200 to 300,000 Yuan	95	69	43	31
Above 300,000 Yuan	21	64	12	36
